# Dopamine Receptor Blockade Attenuates Purinergic P2X4 Receptor-Mediated Prepulse Inhibition Deficits and Underlying Molecular Mechanisms

**DOI:** 10.3389/fncel.2019.00331

**Published:** 2019-07-23

**Authors:** Sheraz Khoja, Liana Asatryan, Michael W. Jakowec, Daryl L. Davies

**Affiliations:** ^1^Titus Family Department of Clinical Pharmacy, School of Pharmacy, University of Southern California, Los Angeles, CA, United States; ^2^Department of Neurology, Keck School of Medicine, University of Southern California, Los Angeles, CA, United States

**Keywords:** ivermectin, P2X4 receptors, dopamine receptors, prepulse inhibition, schizophrenia, DARPP-32

## Abstract

Sensorimotor gating refers to the ability to filter incoming sensory information in a stimulus-laden environment and disruption of this physiological process has been documented in psychiatric disorders characterized by cognitive aberrations. The effectiveness of current pharmacotherapies for treatment of sensorimotor gating deficits in the patient population still remains controversial. These challenges emphasize the need to better understand the biological underpinnings of sensorimotor gating which could lead to discovery of novel drug targets for therapeutic intervention. Notably, we recently reported a role for purinergic P2X4 receptors (P2X4Rs) in regulation of sensorimotor gating using prepulse inhibition (PPI) of acoustic startle reflex. P2X4Rs are ion channels gated by adenosine-5′-triphosphate (ATP). Ivermectin (IVM) induced PPI deficits in C57BL/6J mice in a P2X4R-specific manner. Furthermore, mice deficient in P2X4Rs [P2X4R knockout (KO)] exhibited PPI deficits that were alleviated by dopamine (DA) receptor antagonists demonstrating an interaction between P2X4Rs and DA receptors in PPI regulation. On the basis of these findings, we hypothesized that increased DA neurotransmission underlies IVM-mediated PPI deficits. To test this hypothesis, we measured the effects of D1 and D2 receptor antagonists, SCH 23390 and raclopride respectively and D1 agonist, SKF 82958 on IVM-mediated PPI deficits. To gain mechanistic insights, we investigated the interaction between IVM and dopaminergic drugs on signaling molecules linked to PPI regulation in the ventral striatum. SCH 23390 significantly attenuated the PPI disruptive effects of IVM to a much greater degree than that of raclopride. SKF 82958 failed to potentiate IVM-mediated PPI disruption. At the molecular level, modulation of D1 receptors altered IVM’s effects on dopamine and cyclic-AMP regulated phosphoprotein of 32 kDa (DARPP-32) phosphorylation. Additionally, IVM interacted with the DA receptors antagonists and SKF 82958 in phosphorylation of Ca^2+^/calmodulin kinase IIα (CaMKIIα) and its downstream target, neuronal nitric oxide synthase (nNOS). Current findings suggest an involvement for D1 and D2 receptors in IVM-mediated PPI disruption via modulation of DARPP-32, CaMKIIα and nNOS. Taken together, the findings suggest that stimulation of P2X4Rs can lead to DA hyperactivity and disruption of information processing, implicating P2X4Rs as a novel drug target for treatment of psychiatric disorders characterized by sensorimotor gating deficits.

## Introduction

Sensorimotor gating is an autonomic process of filtering irrelevant sensory information from salient ones in a stimulus-laden environment which is followed by execution of attention-dependent cognitive processes in order to respond to the salient stimuli (Braff and Geyer, 1990; Braff and Light, 2004; Powell et al., 2012). Prepulse inhibition (PPI) of acoustic startle reflex has been identified as an operational measure of sensorimotor gating since it is one of the forms of startle plasticity that resembles the mechanism of sensorimotor gating. PPI of acoustic startle reflex is defined as attenuation of behavioral response to a strong sensory stimulus (pulse) when preceded by a weaker stimulus (prepulse) by 30–500 ms (Graham, 1975; Hoffman and Ison, 1980; Ison and Hoffman, 1983). The observed reduction in response to the pulse stimulus occurs due to activation of inhibitory mechanisms in the central nervous system (CNS) which screens out incoming sensory stimuli until processing of the prepulse stimulus is completed. Hence, PPI serves as a mechanism to avoid interference between distinguishable stimuli (Kumari and Sharma, 2002). The inability to avoid this interference results in significant inundation or overflow of incoming sensory information leading to impairments in attention-dependent cognitive functions (McGhie and Chapman, 1961; Braff, 1993; Braff and Light, 2004). Deficits in PPI have been reported in a wide spectrum of neuropsychiatric disorders that are characterized by cognitive deficits including schizophrenia (Braff and Geyer, 1990; Geyer et al., 2001; Greenwood et al., 2007; Swerdlow et al., 2008; Swerdlow and Light, 2018), bipolar disorder (Perry et al., 2001), manic depressive disorder (Ludewig et al., 2002), Tourette syndrome (Swerdlow et al., 2001b) and autism spectrum disorders such as autism (Perry et al., 2007) and Fragile X syndrome (Frankland et al., 2004).

Previous investigations from our laboratory have reported a role for purinergic P2X4 receptors (P2X4Rs) in modulation of sensorimotor gating (Bortolato et al., 2013; Wyatt et al., 2013; Khoja et al., 2016). P2X4Rs belong to the 7-member P2X superfamily of cation permeable ligand gated ion channels (LGICs) gated by extracellular adenosine-5′-triphosphate (ATP) (North, 2002; North and Verkhratsky, 2006; Khakh and North, 2012). P2X4Rs are abundantly expressed on neurons and glial cells in the CNS (Abbracchio et al., 2009; Khakh and North, 2012). Ivermectin (IVM), a positive modulator of P2X4Rs, disrupted PPI regulation in male C57BL/6J mice and this effect was attenuated in mice deficient in the *p2rx4* gene [i.e., P2X4R knockout (KO) mice] (Bortolato et al., 2013) suggesting that IVM-mediated PPI deficits is P2X4R-dependent. Moreover, male P2X4R KO mice exhibited PPI deficits in comparison to their wildtype (WT) littermates (Wyatt et al., 2013). A possible mechanistic explanation for this behavioral outcome could be the reported increased expression of dopamine (DA) D1 and D2 receptors in the ventral striatum of P2X4R KO mice (Khoja et al., 2016). To further reinforce a role for DA neurotransmission in P2X4R-mediated PPI disruption, DA receptor antagonists significantly ameliorated PPI deficits in P2X4R KO mice (Khoja et al., 2016).

The propensity of IVM to reduce PPI function in C57BL/6J mice is reminiscent of non-selective DA receptor agonists including amphetamine, methamphetamine, cocaine, apomorphine, all of which have been shown to disrupt PPI function in rodents (Ralph et al., 1999; Ralph-Williams et al., 2002, 2003; Doherty et al., 2008). The ability of IVM to mimic pharmacological effects of DA receptor agonists is not surprising considering that we have previously shown that IVM can modulate DA-dependent behaviors and signaling pathways. For instance, IVM potentiated the effects of DA on motor behavior (Khoja et al., 2016). IVM significantly modulated phosphorylation of dopamine and cyclic AMP regulated phosphoprotein of 32 kDa (DARPP-32) (Khoja et al., 2016), which is a critical downstream target regulated by DA receptor agonists and antagonists (Svenningsson et al., 2000, 2003; Nairn et al., 2004; Borgkvist and Fisone, 2007; Bonito-Oliva et al., 2011). The findings from our laboratory and the numerous studies that have described a role for DA in PPI regulation forms the basis for our hypothesis that IVM is causing PPI disruption via activation of DA neurotransmission.

To investigate this hypothesis, we tested the effects of IVM (10 mg/kg, i.p.) on PPI function in the presence of DA receptor antagonists, SCH 23390 (for D1 receptors) and raclopride (for D2 receptors). We also tested the effects of IVM (5 mg/kg, i.p.) in combination with a selective D1 receptor agonist, SKF 82958. Furthermore, to gain mechanistic insights into interaction between IVM and DA receptor antagonists/D1 agonist in PPI regulation, we tested the effects of IVM on phosphorylation of signaling molecules linked to PPI regulation including DARPP-32, Ca^2+^/calmodulin kinase IIα (CaMKIIα) and neuronal nitric oxide synthase (nNOS) in the ventral striatum in the presence of the dopaminergic drugs.

## Materials and Methods

### Animals

Experimentally naïve 3–5 month old male C57BL/6J mice were used for the behavioral experiments and immunoblotting. Mice were either purchased from Jackson Laboratories (Bar Harbor, ME, United States) or procured from our P2X4R KO breeding colony at University of Southern California (USC) that is maintained on a C57BL/6J background. P2X4R KO breeding colony has been described previously (Wyatt et al., 2013, 2014). It was ensured that there were no statistical significant differences in acoustic startle response or PPI% between C57BL/6J mice from Jackson Labs and our breeding colony during the baseline studies. 18–22 mice per treatment group were used for the PPI experiment involving IVM/DA receptor antagonists and 15–16 mice per treatment group were used for the PPI experiment involving IVM/D1 agonist. 3–12 mice per treatment group were used for the immunoblotting experiments. Mice were group housed in cages of 5 in a vivarium maintained at 22°C and 12 h/12 h light: dark cycle and allowed to acclimate to the behavioral testing room for a period of 1 week. All experiments were undertaken as per guidelines established by National Institute of Health (NIH) and approved by Institutional Animal Care and Use Committee (IACUC) at USC. Post completions of behavioral experiments, mice were sacrificed and brain tissues were collected for immunoblotting.

### Materials

Ivermectin (Noromectin, 1% solution, Norbrook Inc, Lenexa, KS, United States) was diluted in 0.9% saline to achieve a concentration that would allow for an injection volume of 0.01 mL/g of body weight. Propylene glycol (Sigma–Aldrich, St. Louis, MO, United States) was used as vehicle control for IVM. SCH 23390, raclopride and SKF 82958 (Sigma-Aldrich, St. Louis, MO, United States) were diluted in 0.9% saline to achieve concentrations of 0.2 mg/mL, 0.6 mg/mL, and 0.02 mg/mL respectively that would allow an injection volume of 0.005 mL/g of body weight.

### Acoustic Startle and PPI of Acoustic Startle Reflex

#### Apparatus

Acoustic Startle reflex and PPI session were tested as described previously (Wyatt et al., 2013; Khoja et al., 2016). The apparatus used for detection of acoustic startle and PPI (San Diego Instruments, San Diego, CA, United States) consisted of a standard cage placed in sound attenuated chambers with fan ventilation. Each cage consisted of a Plexiglass cylinder mounted on a piezoelectric accelerometric platform connected to an analog-digital converter. Background noises and acoustic bursts were conveyed by two separate speakers, each one oriented in a way that produced variation of 1 Db across startle cage. Both speakers and startle cages were connected to the main PC, which detected all the chamber variables. Before baseline or testing session, the machine was calibrated using digital sound level meter.

#### Startle and PPI Session

In the baseline session, mice were exposed to background noise of 70 Db and after an acclimation period of 5 min, were presented with 12 ms of 40 trials of 115 Db interposed with three trials of a 82 Db prestimulus preceding the 115 Db by 100 ms. Acoustic startle magnitude and PPI % were equivalent across all treatment groups. On the testing day, each mouse was exposed to an acclimation period of 5 min which comprised of background sound 70 Db, which continued for remainder of session. Each session consisted of three consecutive blocks of trials. During the first and third blocks, mice were exposed to five pulse alone trials of 115 Db. In the second block, mice were exposed to pseudorandom sequence of 50 trials, which consisted of 12 pulse alone trials, 30 trials of pulse preceded by 73, 76, or 82 Db (defined as PPI 3, PPI 6, and PPI 12 respectively; 10 for each level of prepulse loudness) and 8 no stimulus trials, wherein there was only background sound without any prepulse or pulse stimuli. Inter trial intervals were chosen between 10 and 15 s. PPI% was calculated as 100-[(mean startle amplitude for pre-pulse pulse trials/mean startle amplitude for pulse alone trials) × 100].

Ivermectin (or vehicle) (10 mg/kg, i.p. for DA receptor antagonist PPI experiment and 5 mg/kg, i.p. for D1 agonist PPI experiment) was injected 8 h prior to behavioral testing. This time-point is based on previous studies where we have shown correlation between pharmacological effects and maximal concentration (Cmax) as well as time to reach maximum concentration (Tmax) (Yardley et al., 2012; Bortolato et al., 2013). SCH 23390 (1 mg/kg, i.p.), raclopride (3 mg/kg, i.p.) or SKF 82958 (0.1 mg/kg, i.p.) was injected 10 min prior to behavioral testing.

### Western Immunoblotting

#### Drug Treatments and Tissue Preparation

Ivermectin (or vehicle) was administered 8 h and 10 min prior to euthanasia. SCH 23390 (1 mg/kg, i.p.), raclopride (3 mg/kg, i.p.) or SKF 82958 (0.1 mg/kg) was injected 10 min prior to 8th h of IVM/vehicle administration and so, animals were euthanized 20 min post administration of dopaminergic drugs. The PPI testing duration is approximately 20 min and dopaminergic drugs were injected 10 min before the PPI session. Therefore, we chose to euthanize the mice 10 min post the 8th h of IVM/vehicle administration so that there is sufficient amount of time for the dopaminergic drugs to have an effect on IVM-mediated signaling pathways in the ventral striatum.

Ventral striatum was dissected out as per the landmarks described in the mouse brain atlas (Franklin and Paxinos, 2007). The brain tissues were homogenized in a buffer containing 50 mM tris-HCl pH (7.4), 150 mM NaCl, 0.5% sodium deoxycholate, 1% Triton-X-100, 0.1% SDS, 1% proteinase inhibitor cocktail (EMD Millipore, Temecula, CA, United States) and a cocktail of phosphatase inhibitors (1 mM sodium pyrophosphate, 10 mM sodium fluoride, 0.5 mM sodium orthovanadate, 10 mM β-glycerol phosphate, 1 μM microcysteine). In addition to these inhibitors, the homogenates were treated with 1% phosphatase inhibitors sets 1 and 2 (EMD Millipore, Temecula, CA, United States). Protein content was determined by BCA assay (Thermo Scientific, Rockville, IL, United States).

#### Immunoblotting Procedure

Ventral striatal tissues ran on 10% SDS-PAGE gels (50 μg/lane). Due to low volume of certain samples, protein concentrations of 40 μg/lane (for CaMKIIα) and 30 μg/lane (for nNOS) ran on some of the gels. The samples were then transferred onto PVDF membranes using the Trans-turbo blot system (Bio-Rad, Hercules, CA, United States). Non-specific binding was blocked by incubating the membranes with 5% non-fat dry milk (Bio-Rad, Hercules, CA, United States) followed by incubation with primary antibodies overnight at 4°C. Incubation with loading control antibodies was performed at room temperature for 8 min. The phospho-specific antibodies used for immunoblotting were rabbit-anti-phospho-Thr34-DARPP-32 (1:350; EMD Millipore, Temecula, CA, United States), rabbit- anti-phospho-Thr286-CamKII (1:500; Cell Signaling Technology, Beverly, MA, United States), rabbit-anti-phospho-Ser897-nNOS (1:500; Abcam, Cambridge, United Kingdom). Rabbit polyclonal antibodies raised against DARPP-32 (1:1000; EMD Millipore, Temecula, CA, United States), CaMKIIα (1:1000; Cell Signaling Technology, Beverly, MA, United States) and nNOS (1:1000; Abcam, Cambridge, United Kingdom) which are not phosphorylation specific, were used to determine total amounts of protein. Mouse monoclonal antibodies raised against β-actin (1:20,000; Sigma-Aldrich, St. Louis, MO, United States), α-tubulin (1:20,000; EMD Millipore, Temecula, CA, United States) and α-vinculin (1:1000; Sigma-Aldrich, St. Louis, MO, United States) were used as loading control antibodies. Primary antibody incubation was followed by incubation with secondary antibodies including goat-anti-mouse and anti-rabbit antibody (Bio-Rad, Hercules, CA, United States) for 1 h at room temperature. After incubation with Clarity western plus substrate (Bio-Rad, Hercules, CA, United States), bands were visualized using chemiluminescence imaging on the ChemiDoc system (Bio-Rad, Hercules, CA, United States). Quantification of bands was performed using ImageJ software (NIH, Bethesda, MD, United States).

### Statistical Analyses

To evaluate the effects of dopaminergic drugs on IVM-mediated PPI regulation, we performed repeated measures three way ANOVA using prepulse intensity as repeated variable and IVM treatment and SCH 23390/raclopride/SKF 82958 pre-treatments as independent variables, followed by Tukey’s *post hoc* test for multiple comparisons. Non-repeated measures two way ANOVA followed by Tukey’s *post hoc* test was used to analyze interactions between IVM and dopaminergic drugs in regulation of acoustic startle magnitude.

For the Western blotting analyses, the average densitometry values of vehicle treated samples was arbitrarily normalized to 1 and the samples from drug treatment groups were normalized by dividing each value by the average of vehicle treated samples. The degree of phosphorylation was calculated as ratio of normalized values of phosphorylated form to total form of protein and expressed as mean ± SEM. It was ensured that the densitometry values of treatment groups were normalized to their respective controls prior to combining the data from different membranes for statistical analyses. Non-repeated measures two way ANOVA with Tukey’s *post hoc* test was used to investigate the interaction between IVM and the dopaminergic drugs in regulation of total levels and phosphorylation of various signaling molecules. Significance was set at *P* < 0.05. All data was analyzed using GraphPad Prism software (San Diego, CA, United States).

## Results

### Pharmacological Blockade of D1 as Well as D2 Receptors Significantly Attenuated IVM-Mediated PPI Disruption in Mice

**FIGURE 1:**
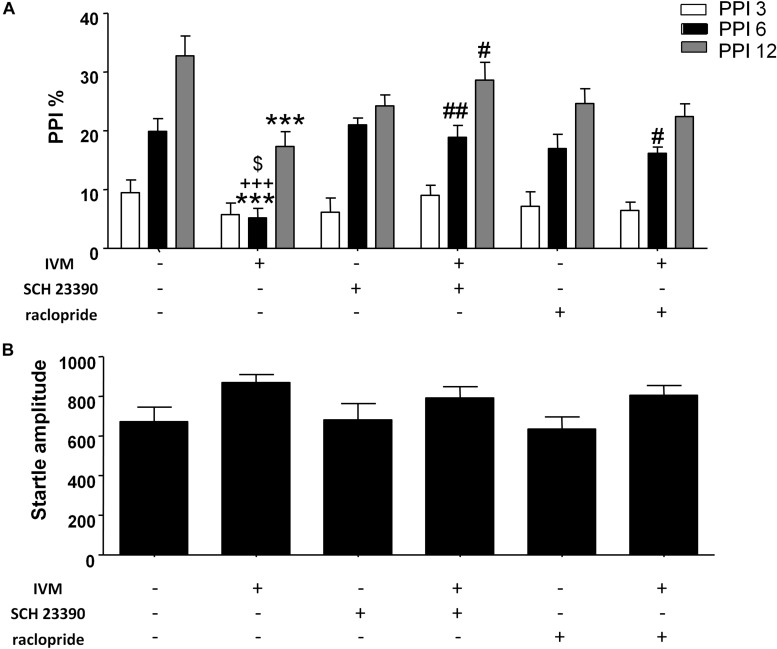
Ivermectin (10 mg/kg) significantly disrupted PPI function in mice at PPI 6 and PPI 12. Co-treatment with SCH 23390 (1 mg/kg) significantly reversed IVM-mediated PPI disruption at both these prepulse intensities. Co-treatment with raclopride (3 mg/kg) reversed IVM-mediated PPI deficits at PPI 6, but not at PPI 12 **(A)**. Neither of the DA receptor antagonists significantly impacted IVM-mediated effects on startle magnitude **(B)**. IVM significantly affected startle magnitude. Values represent mean PPI % or startle amplitude ± SEM from 18 to 22 mice per treatment group. ^∗∗∗^*P* < 0.001 versus control group. ^#^*P* < 0.05, ^##^*P* < 0.01 versus IVM-treated mice. ^+++^*P* < 0.001 versus SCH 23390-treated mice and ^$^*P* < 0.05 versus raclopride-treated mice, Tukey’s multiple comparison test.

The impact of D1 and D2 receptor antagonism on IVM-mediated PPI regulation was assessed. There was a significant effect of prepulse intensity [*F*(2,152) = 81.82, *p* < 0.001] on the account of a gradual increase in PPI% as the prepulse stimulus intensity increases. In agreement with our previous findings (Bortolato et al., 2013), administration of IVM (10 mg/kg, i.p.) significantly disrupted PPI function [*F*(1,76) = 8.983, *p* < 0.01]. Treatment with D1 antagonist, SCH 23390, tended to affect PPI function as indicated by a non-significant trend toward effect of pre-treatment on PPI% [*F*(1,76) = 3.306, *p* = 0.0741]. In both cases, there was a significant treatment × prepulse intensity interaction [*F*(2,152) = 4.040, *p* < 0.05] as well as a pre-treatment × prepulse intensity interaction [*F*(2,152) = 3.818, *p* < 0.05] suggesting that the effects of IVM and SCH 23390 on PPI% were dependent upon the prepulse intensity. Most importantly, SCH 23390 significantly antagonized IVM-mediated PPI dysfunction as indicated by a significant treatment × pre-treatment interaction [*F*(1,76) = 16.48, *p* < 0.001]. Tukey’s *post hoc* test revealed that IVM significantly reduced PPI% in comparison to its vehicle group at PPI 6 (*q* = 6.447, *p* < 0.001) and PPI 12 (*q* = 6.735, *p* < 0.001) (Figure 1A). IVM-mediated PPI disruption at both these prepulses was significantly reversed upon co-treatment with SCH 23390 (*q* = 6.05, *p* < 0.01 for PPI 6 and *q* = 4.982, *p* < 0.05 for PPI 12) (Figure 1A). Tukey’s *post hoc* test also revealed that IVM significantly reduced PPI% in relation to SCH 23390-treated mice at PPI 6 (*q* = 6.647, *p* < 0.001) and co-treatment with IVM and SCH 23390 normalized IVM-induced PPI deficits (Figure 1A). In the context of acoustic startle reactivity, IVM significantly affected acoustic startle magnitude [*F*(1,76) = 5.62, *p* < 0.05] without any interaction with SCH 23390, indicating that IVM modulated startle response independent of D1 receptors (Figure 1B).

There was no significant effect of D2 antagonist, raclopride, on PPI function [*F*(1,73) = 0.1442, *p* > 0.1] indicating the inability of raclopride to independently modulate PPI. Similar to SCH 23390 treatment, raclopride significantly blocked IVM-mediated PPI disruption as indicated by a significant treatment × pre-treatment interaction [*F*(1,73) = 11.51, *p* < 0.01]. Co-treatment of IVM with raclopride significantly reversed the disruptive effects of IVM at PPI 6 (*q* = 4.738, *p* < 0.05) but, unlike SCH 23390, was unable to impede IVM-mediated PPI disruption at PPI 12 (*q* = 2.197, *p* > 0.1) (Figure 1A). Similar to SCH 23390-treated mice, administration of IVM significantly decreased PPI% in comparison to raclopride-treated mice at PPI 6 (*q* = 5.163, *p* < 0.05) and this effect was restored upon co-treatment with IVM and raclopride (Figure 1A). Noticeably, mice co-treated with IVM and raclopride tended to have lower PPI % in comparison to mice treated with vehicle for IVM at PPI 12 (*q* = 4.571, *p* = 0.0616), suggesting the failure of raclopride to fully reverse the disruptive effects of IVM at PPI 12 (Figure 1A). Similarly, raclopride did not alter the significant effects of IVM on startle magnitude as revealed by a non-significant treatment × pre-treatment interaction (Figure 1B).

### D1 Receptor Activation Did Not Potentiate the Effects of IVM on PPI Function

**FIGURE 2:**
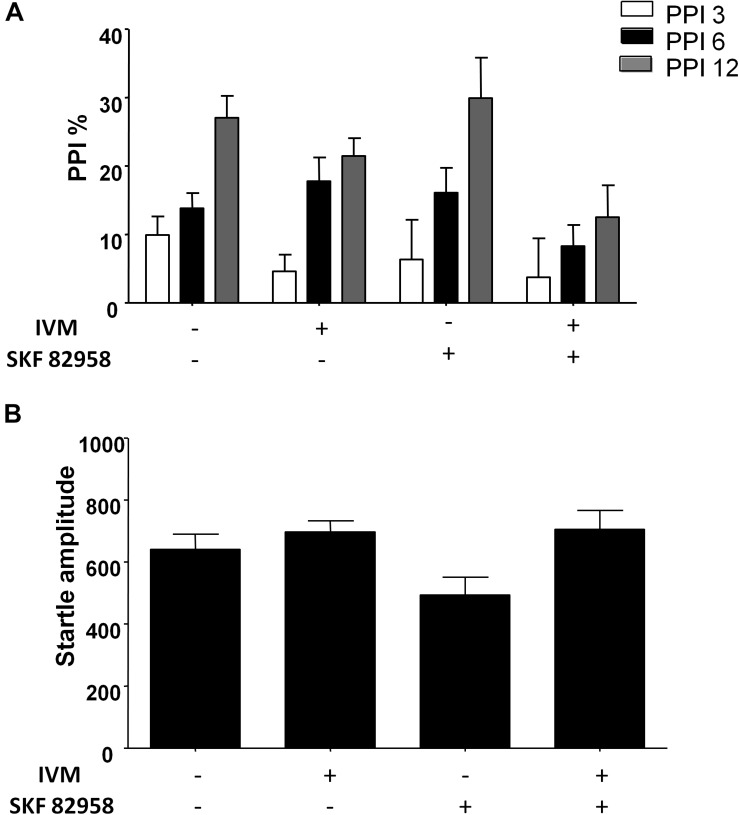
D1 receptor stimulation by SKF 82958 (0.1 mg/kg) failed to potentiate IVM (5 mg/kg)-mediated PPI disruption. **(A)** There was no significant interaction between IVM and SKF 82958 in regulation of startle magnitude. **(B)** Values represent mean PPI % or startle amplitude ± SEM from 15 to 16 mice per treatment group.

There was a non-significant trend toward effect of IVM treatment (5 mg/kg, i.p.) on PPI function [*F*(1,57) = 3.626, *p* = 0.0619] and treatment × prepulse intensity interaction tended toward statistical significance [*F*(2,114) = 2.504, *p* = 0.0863]. Treatment with D1 agonist, SKF 82958, did not have any significant effect on PPI function [*F*(1,57) = 0.9509, *p* > 0.1] (Figure 2A). Furthermore, there was no synergistic interaction between IVM and SKF 892958 on PPI% as indicated by a non-significant treatment × pre-treatment interaction [*F*(1,57) = 1.304, *p* > 0.1] (Figure 2B).

### Pharmacological Modulation of Either D1 or D2 Receptors Significantly Altered IVM-Induced Changes in DARPP-32 Phosphorylation in the Ventral Striatum

**FIGURE 3:**
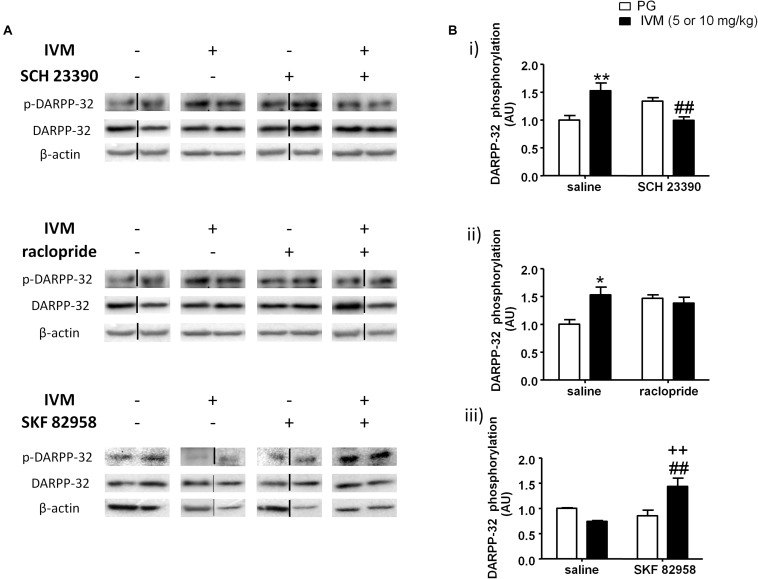
Blockade of D1 receptors by SCH 23390 significantly reversed IVM (10 mg/kg)-mediated increase in DARPP-32 phosphorylation **(A,Bi)** in the ventral striatum. Blockade of D2 receptors by raclopride antagonized, but did not reverse, IVM (10 mg/kg)-mediated changes in DARPP-32 phosphorylation **(A,Bii)**. Stimulation of D1 receptors by SKF 82958 significantly interacted with IVM (5 mg/kg) in a synergistic manner **(A,Biii)**. The average of densitometry value of control group was arbitrarily normalized to 1. The treatment groups were normalized by dividing each value by the average of the control group. The data is presented as fold change of treatment group versus control group in that membrane. Values represent mean ± SEM from 4 to 10 mice per treatment group. Two representative bands from each treatment group from different gels are shown. IVM alone (or vehicle) images were used twice for the IVM/SCH 23390 and IVM/raclopride blots since both the DA receptor antagonist treatment groups (with or without IVM) ran on the same gel. Black solid lines between the bands indicate that the bands were cropped and spliced together either from different gels or non-consecutive lanes. Full scan of gel images can be found in the Supplementary Figures S1–S3. ^*^P < 0.05; ^∗∗^*P* < 0.01 versus control group; ^##^*P* < 0.01 versus IVM –treated mice (5 and 10 mg/kg); ^++^*P* < 0.01 versus SKF 82958-treated mice, Tukey’s multiple comparison test.

We investigated the interaction between IVM and SCH 23390/raclopride on DARPP-32 phosphorylation in the ventral striatum on the basis of a critical role for DARPP-32 in PPI regulation (Svenningsson et al., 2003; Kuroiwa et al., 2012; Yabuki et al., 2013). There was no significant effect of IVM (10 mg/kg) or SCH 23390 treatment on DARPP-32 phosphorylation but there was a significant treatment × pre-treatment interaction suggesting that SCH 23390 altered the effects of IVM on DARPP-32 phosphorylation [*F*(1,29) = 18.16, *p* < 0.001]. Tukey’s *post hoc* test confirmed that IVM significantly increased DARPP-32 phosphorylation in comparison to its vehicle (*q* = 5.222, *p* < 0.01) and this effect was significantly reversed upon co-treatment with SCH 23390 (*q* = 5.658, *p* < 0.01) (Figures 3A,Bi). Moreover, there was no significant treatment × pre-treatment interaction in regulation of total DARPP-32 levels indicating that SCH 23390 significantly modulated the effects of IVM on DARPP-32 phosphorylation without any impact on total protein levels.

On the other hand, raclopride did not exhibit any significant effect on DARPP-32 phosphorylation. Similar to SCH 23390, raclopride significantly antagonized IVM-induced increase in DARPP32 phosphorylation as indicated by a significant treatment × pre-treatment interaction [*F*(1,23) = 5.419, *p* < 0.05]. Tukey’s *post hoc* test revealed a significant increase in DARPP-32 phosphorylation upon treatment with IVM alone (*q* = 4.693, *p* < 0.05) and co-administration with raclopride blocked the effects of IVM on DARPP-32 phosphorylation in relation to raclopride-treated mice (*q* = 0.5589, *p* > 0.1) (Figures 3A,Bii). However, co-administration with raclopride failed to reverse IVM-mediated increase in DARPP-32 phosphorylation (Figures 3A,Bii). Finally, similar to SCH 23390, raclopride does not modulate IVM’s effects on total DARPP-32 levels as revealed by a non-significant treatment × pre-treatment interaction, indicating that the interaction between IVM and raclopride is specific to phosphorylation and not to total protein levels.

Considering that blockade of DA D1 receptors impedes IVM-mediated changes in DARPP-32 phosphorylation, we next evaluated whether DA D1 receptor activation can potentiate DARPP-32 phosphorylation in presence of IVM (5 mg/kg). There was a significant effect of SKF 82958 [*F*(1,20) = 5.319, *p* < 0.05] but not that of IVM treatment on DARPP-32 phosphorylation. In support of our hypothesis, SKF 82958 significantly up-regulated DARPP-32 phosphorylation in the presence of IVM as revealed by a significant treatment × pre-treatment interaction [*F*(1,20) = 12.58, *p* < 0.01]. Tukey’s *post hoc* test confirmed that co-administration of IVM and SKF 82958 significantly up-regulated DARPP-32 phosphorylation in relation to IVM-treated mice (*q* = 5.853, *p* < 0.01), SKF 82958-treated mice (*q* = 5.373, *p* < 0.01) and tended to increase DARPP-32 phosphorylation in relation to mice treated with vehicle for IVM (*q* = 3.664, *p* = 0.0759) (Figures 3A,Biii). Finally, there were no significant effects of both IVM and SKF 82958 treatments on total DARPP-32 levels and the interaction between the two drugs was not significant.

### Pharmacological Modulation of D1 and D2 Receptors Significantly Altered IVM-Mediated Changes in CaMKIIα Phosphorylation in the Ventral Striatum

**FIGURE 4:**
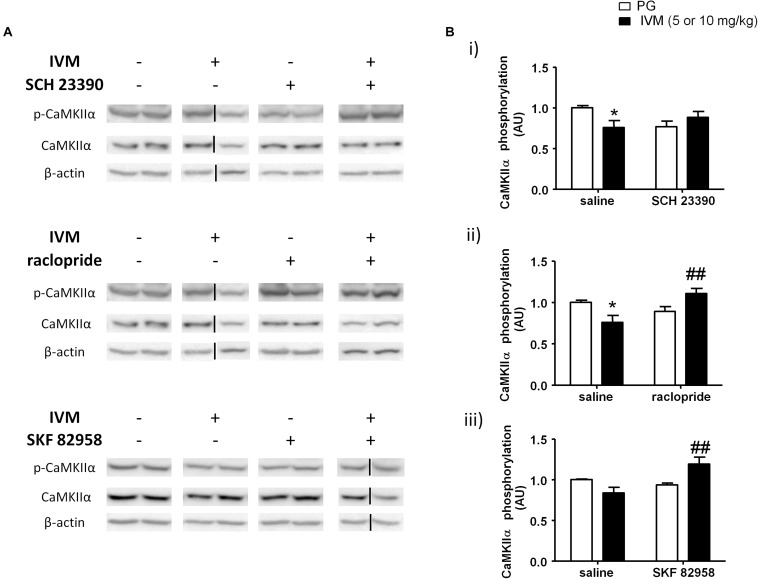
IVM (10 mg/kg)-mediated decrease in CaMKIIα phosphorylation was significantly impeded by SCH 23390 **(A,Bi)** but reversed by raclopride **(A,Bii)** in the ventral striatum. IVM (5 mg/kg) synergistically interacted with SKF 82958 in regulation of CaMKIIα phosphorylation **(A,Biii)** in the ventral striatum. Details of normalization and analyses are presented in Figure 3. Values represent mean ± SEM from 3 to 11 mice per treatment group. Two representative bands from each treatment group are shown. IVM alone (or vehicle) images were used twice for the IVM/SCH 23390 and IVM/raclopride blots since both the DA receptor antagonist treatment groups (with or without IVM) ran on the same gel. Black solid lines indicated that bands were cropped and spliced from non-consecutive lanes or different gels. Full scan of gel images can be found in the Supplementary Figures S4, S5. ^*^P < 0.05 versus control group; ^##^*P* < 0.01 versus IVM-treated mice (5 and 10 mg/kg), Tukey’s multiple comparison test.

CaMKIIα has been previously linked to regulation of information processing (Coultrap and Bayer, 2012) and pharmacological agents that are known to induce PPI deficits have been reported to cause alterations in CaMKIIα phosphorylation (Suemaru et al., 2000; Molteni et al., 2008; Cui et al., 2009). On the basis that P2X (Leon et al., 2006) and DA signaling (Anderson et al., 2008; Ng et al., 2010) have been associated with CaMKIIα regulation, we tested to determine if DA receptor modulation can regulate IVM-mediated effects on CaMKIIα phosphorylation. There was neither any significant effect of IVM (10 mg/kg) nor SCH 23390 treatment on CaMKIIα phosphorylation. However, SCH 23390 significantly blocked the effects of IVM on CaMKIIα phosphorylation as revealed by a significant treatment × pre-treatment interaction. [*F*(1,31) = 6.343, *p* < 0.05] with Tukey’s *post hoc* test confirming that IVM significantly decreased CaMKIIα phosphorylation in relation to the vehicle group (*q* = 3.852, *p* < 0.05) (Figures 4A,Bi). Furthermore, SCH 23390 tended to decrease CaMKIIα phosphorylation in comparison to the control group (*q* = 3.423, *p* = 0.0940). Co-treatment of IVM and SCH 23390 blocked the effects of IVM on CaMKIIα phosphorylation in comparison to SCH 23390-treated mice (*q* = 1.465, *p* > 0.1) but failed to completely reverse IVM-induced decrease in CaMKIIα phosphorylation (Figures 4A,Bi). With respect to total protein levels, there was no significant effect of either IVM or SCH 23390 treatment on total CaMKIIα levels but the treatment × pre-treatment interaction tended toward statistical significance [*F*(1,31) = 2.931, *p* = 0.0969].

Raclopride did not exhibit any significant effect on CaMKIIα phosphorylation. However, there was a significant treatment × pre-treatment interaction [*F*(1,28) = 10.39, *p* < 0.01] suggesting that raclopride antagonized IVM-mediated effects on CaMKIIα phosphorylation. Tukey’s *post hoc* test confirmed that IVM significantly decreased CaMKIIα phosphorylation (*q* = 4.089, *p* < 0.05) which was significantly reversed upon co-treatment with raclopride (*q* = 5.116, *p* < 0.01) (Figures 4A,Bii). In contrast to SCH 23390, there was a significant effect of raclopride on total CaMKIIα levels [*F*(1,28) = 4.502, *p* < 0.05] without any significant interaction with IVM suggesting that raclopride altered total protein levels independent of IVM treatment.

DA D1 receptor activation by SKF 82958 induced independent changes in CaMKIIα phosphorylation [*F*(1,14) = 5.053, *p* < 0.05] that was significantly potentiated in the presence of IVM (5 mg/kg) as indicated by a significant treatment × pre-treatment interaction [*F*(1,14) = 10.55, *p* < 0.01]. Tukey’s *post hoc* test detected that co-treatment of IVM and SKF 82958 significantly increased CaMKIIα phosphorylation in comparison to IVM-treated mice (*q* = 5.936, *p* < 0.01) (Figures 4A,Biii). Moreover, co-administration of IVM and SKF 82958 tended to enhance phosphorylation in relation to SKF 82958-treated mice (*q* = 3.747, *p* = 0.0796) (Figures 4A,Biii). Furthermore, there was a significant effect of both IVM [*F*(1,14) = 9.404, *p* < 0.01] and SKF 82958 [*F*(1,14) = 9.723, *p* < 0.01] treatments on total CaMKIIα levels without any significant interaction between the two treatments suggesting the inability of SKF 82958 to modulate IVM-induced changes in total CaMKIIα levels.

### IVM Significantly Interacted With DA Receptor Antagonists/D1 Agonist in Regulation of nNOS Phosphorylation in the Ventral Striatum

**FIGURE 5:**
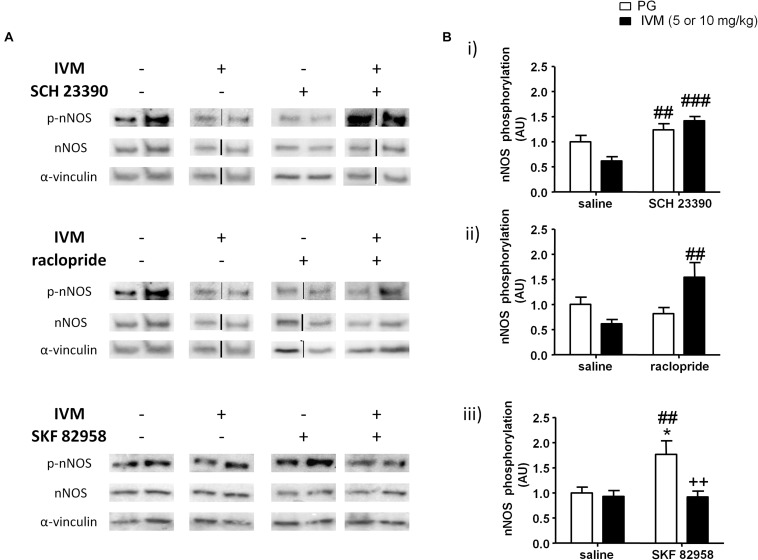
SCH 23390 **(A,Bi)** and raclopride **(A,Bii)** significantly increased nNOS phosphorylation in presence of IVM (10 mg/kg) in the ventral striatum. IVM (5 mg/kg) significantly attenuated SKF 82958-induced increase in nNOS phosphorylation **(A,Biii)**. Details of normalization and analyses are presented in Figure 3. Values represent mean ± SEM from 4 to 12 mice per treatment group. Two representative bands from each treatment group are shown. IVM alone (or vehicle) images were used twice for the IVM/SCH 23390 and IVM/raclopride blots since both the DA receptor antagonist treatment groups (with or without IVM) ran on the same gel. Black solid lines between bands indicate that bands were cropped and spliced together from non-consecutive lanes or from different gels. Full scan of gel images can be found in the Supplementary Figures S6, S7. ^*^P < 0.05 versus control group; ^##^*P* < 0.01, ^###^*P* < 0.001 versus IVM-treated mice (5 and 10 mg/kg); ^++^*P* < 0.01 versus SKF 82958-treated mice, Tukey’s multiple comparison test.

P2X4Rs have been previously implicated in regulation of NOS activity and NO release in cardiac myocytes (Yang and Liang, 2012; Yang et al., 2015). Considering that NOS inhibitors have been reported to ameliorate PPI impairments induced by psychostimulants and the involvement of NO in PPI regulation (Wiley, 1998; Klamer et al., 2004; Fejgin et al., 2008; Issy et al., 2011, 2014), we hypothesized that nNOS could be one of potential substrates that underlies the antagonistic interaction between IVM and DA receptor antagonists in PPI regulation. There was a significant effect of SCH 23390 [*F*(1,21) = 19.96, *p* < 0.001], but not that of IVM (10 mg/kg), treatment on nNOS phosphorylation. In addition to regulating nNOS phosphorylation independently, SCH 23390 altered the effects of IVM as indicated by a significant treatment × pre-treatment interaction [*F*(1,21) = 5.96, *p* < 0.05]. Tukey’s *post hoc* test detected a non-significant trend toward decrease in nNOS phosphorylation upon IVM treatment alone in comparison to the vehicle group (*q* = 3.905, *p* = 0.0528) which was significantly reversed upon co-administration with SCH 23390 (*q* = 7.17, *p* < 0.001) (Figures 5A,Bi). Furthermore, treatment with SCH 23390 alone significantly increased nNOS phosphorylation in comparison to IVM-treated mice (*q* = 5.146, *p* < 0.01) (Figures 5A,Bi). In regards to total protein levels, there was neither any significant effect of both the treatments nor was there any significant interaction suggesting that SCH 23390 altered effects of IVM on nNOS phosphorylation without any effect on total protein levels.

Similar to SCH 23390 treatment, administration of raclopride alone significantly affected nNOS phosphorylation [*F*(1,20) = 4.839, *p* < 0.05] and was able to significantly alter IVM-mediated effects on NOS phosphorylation as revealed by a significant treatment × pre-treatment interaction [*F*(1,20) = 10.65, *p* < 0.01]. Tukey’s *post hoc* test confirmed that co-treatment of IVM and raclopride significantly increased nNOS phosphorylation in comparison to IVM-treated mice (*q* = 5.741, *p* < 0.01) (Figures 5A,Bii). There was a significant effect of IVM [*F*(1,20) = 4.996, *p* < 0.05], but not that of raclopride, treatment on total nNOS levels. Additionally, the interaction between IVM and raclopride for total nNOS levels tended toward statistical significance [*F*(1,20) = 3.347, *p* = 0.0823].

In contrast to a high dose (10 mg/kg) of IVM; there was a significant effect of 5 mg/kg IVM on nNOS phosphorylation [*F*(1,33) = 8.22, *p* < 0.01]. Additionally, D1 receptor activation by SKF 82958 significantly increased nNOS phosphorylation alone [*F*(1,33) = 5.524, *p* < 0.05] and this effect was significantly attenuated by IVM as indicated by a significant treatment × pre-treatment interaction [*F*(1,33) = 5.998, *p* < 0.05]. Tukey’s *post hoc* test detected a significant increase in nNOS phosphorylation upon SKF 82958 treatment alone in comparison to the control group (*q* = 4.656, *p* < 0.05) as well as IVM-treated mice (*q* = 4.919, *p* < 0.01) (Figures 5A,Biii). Most importantly, co-treatment with IVM and SKF 82958 significantly reduced nNOS phosphorylation in comparison to SKF 82958-treated mice (*q* = 5.491, *p* < 0.01) (Figures 5A,Biii). Finally, there was neither any significant effect of IVM or SKF 82958 treatment on total nNOS levels nor was there any significant interaction indicating that IVM was able to modulate SKF 82958-mediated changes in nNOS phosphorylation without any impact on total protein levels.

## Discussion

The current study investigated the mechanisms underlying IVM induced PPI disruption in C57BL/6J mice. PPI deficits induced upon P2X4R potentiation by IVM were significantly attenuated to a greater extent via antagonism of D1 than D2 receptors, implicating a more essential role for D1 than D2 receptors in IVM-mediated PPI dysfunction. Additionally, neither of the DA receptor antagonists modified the effects of IVM on startle magnitude suggesting that DA antagonists were able to restore IVM-induced information processing deficits without having any impact on auditory function. The findings from the DA receptor antagonist experiments are in agreement with previous investigations that have reported a more important role for D1 receptors than D2 receptors in PPI regulation in mice (Ralph-Williams et al., 2002, 2003; Ralph and Caine, 2005). For instance, non-selective DA receptor agonists such as apomorphine, cocaine or pergolide have been reported to disrupt PPI function in a D1 receptor-specific manner (Ralph-Williams et al., 2002, 2003; Doherty et al., 2008). Selective D1 agonists such as SKF 82958, SKF 81297 and dihydrexidine have also been shown to disrupt PPI functioning in C57BL/6J and 129S6 mice (Ralph-Williams et al., 2003; Ralph and Caine, 2005) at doses lower than those required to induce similar behavioral effects with rats (Wan et al., 1996; Swerdlow et al., 2000). Moreover, findings from our laboratory reported that D1 receptor antagonism rescued PPI dysfunction in P2X4R KO mice (Khoja et al., 2016). Taken together, the current body of evidence strongly suggests that D1 receptors have a prominent role in PPI regulation in mice.

In addition to the importance of D1 receptors in PPI regulation with mice; the role of D2 receptors cannot be disregarded based on our findings where raclopride significantly blocked IVM-mediated PPI dysfunction. Moreover, indirect DA receptor agonists including amphetamine and cocaine can disrupt PPI function via D2 receptor-dependent manner in mice (Ralph et al., 1999; Ralph-Williams et al., 2002; Doherty et al., 2008). In addition, multiple genetic knockout mouse models for dopamine transporter (DAT) (Ralph et al., 2001), disrupted in schizophrenia complex (DISC) (Lipina et al., 2010) and trace amine receptor-1 (TA1) (Wolinsky et al., 2007) exhibited PPI deficits that were linked to aberrant D2 receptor activity. In agreement with pharmacological and genetic findings, our laboratory has previously demonstrated that PPI deficits in P2X4R KO mice were also reversed by raclopride, in addition to SCH 23390, indicating that D2 receptors can be involved in PPI dysfunction in P2X4R KO mice (Khoja et al., 2016).

The findings from our IVM/P2X4R KO PPI investigations (and others) suggest a plausible D1–D2 receptor interaction in PPI regulation in mice. The argument for this hypothesis is reinforced by previous reports showing that antagonism of either D1 or D2 receptors can negate PPI deficits induced by D1 agonists in mice (Ralph and Caine, 2005). For example, administration of either D2 antagonist eticlopride or D1 antagonist SCH 39166 significantly restored SKF 82958-mediated PPI deficits in C57BL/6J mice (Ralph and Caine, 2005). A plausible explanation for this outcome is that activation of D1 receptors alone might not be sufficient enough to disrupt PPI function in rats but the ability of D2 receptors to induce PPI dysfunction is dependent upon the tonic activity of D1 receptors. Hence, based on our current findings, it appears that D1 receptors might be interacting with D2 receptors in the modulation of IVM-mediated PPI alterations and the tendency of raclopride to block IVM’s behavioral effects might be dependent upon tonic activity of D1 receptors in C57BL/6J mice.

**FIGURE 6:**
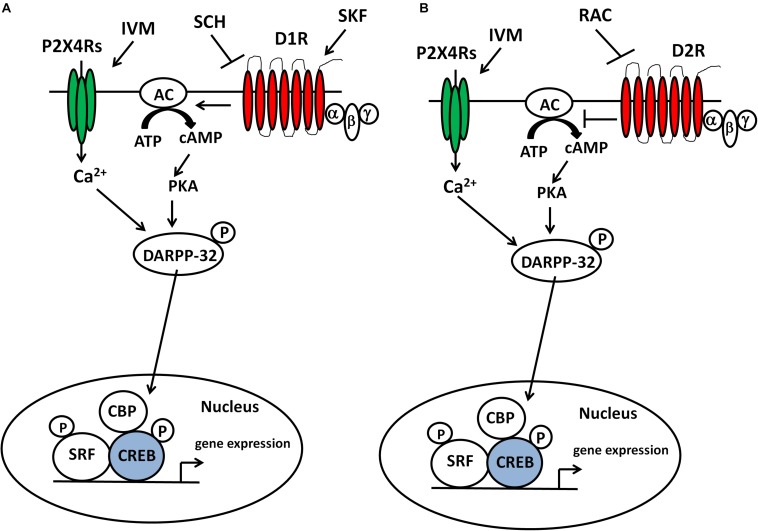
IVM modulated DARPP-32 phosphorylation via P2X4R potentiation in a D1 receptor-dependent manner in the ventral striatum. Inter-dependence between P2X4Rs and D1 receptors in DARPP-32 phosphorylation may be relevant to regulation of transcription factors (e.g., CREB) and subsequent gene expression in the ventral striatum and DA-dependent behaviors such as sensorimotor gating **(A)**. The ability of raclopride to block IVM-mediated changes at behavioral and cellular level indicates a plausible interaction between P2X4Rs and D2 receptors in regulation of DA-dependent behaviors and signaling pathways **(B)**.

To gain insights into the molecular mechanisms by which IVM can induce PPI dysfunction, we investigated its interaction with dopaminergic drugs on DARPP-32 phosphorylation in the ventral striatum. Our findings suggest that positive modulation of P2X4Rs increased DARPP-32 phosphorylation in a D1 receptor dependent manner (Figure 6A). Although, raclopride was able to prevent IVM from further increasing DARPP-32 phosphorylation, it failed to reverse IVM-mediated increase in DARPP-32 phosphorylation, suggesting that the actions of IVM on DARPP-32 are D1-dependent. Enhanced phosphorylation of DARPP-32 at Thr 34 has been linked to PPI deficits mediated by various psychostimulants such as amphetamine, phencyclidine (PCP), lysergic acid diethylamide (LSD) and that these effects were significantly diminished in genetically modified mice wherein DARPP-32 phosphorylation at Thr34 is compromised (Svenningsson et al., 2003). D1 receptor activation on the striatonigral neurons (D1-expressing neurons) of the basal ganglia circuitry stimulates the cAMP/protein kinase-A (PKA) pathway which leads to increased phosphorylation of DARPP-32 at Thr34 (Svenningsson et al., 2004; Girault, 2012) (Figure 6) and possibly manifestation of PPI deficits in mice as demonstrated by selective D1 agonists (Ralph-Williams et al., 2003; Ralph and Caine, 2005). Taking these findings into perspective, the reversal of IVM-mediated changes in DARPP-32 phosphorylation by SCH 23390 may underlie the attenuation of IVM-mediated PPI deficits. Moreover, the failure of raclopride to reverse IVM-induced increase in DARPP-32 phosphorylation might explain the discrepancy between SCH 23390 and raclopride in restoration of IVM-induced PPI deficits. However, this inference cannot be supported by the current evidence that SKF 82958 failed to elicit PPI dysfunction in presence of IVM despite the synergistic interaction between SKF 82958 and IVM in augmentation of DARPP-32 phosphorylation in the ventral striatum. This discrepancy can be attributed to the notion that DARPP-32 alone cannot mediate PPI function considering that multiple other neural substrates may contribute to the alleviation of IVM-induced PPI disruption by SCH 23390. Furthermore, the high dose of SCH 23390 used in this study may involve pharmacological activity at other receptor targets such as agonism at 5-HT2C receptors (Briggs et al., 1991; Millan et al., 2001). Notably, 5-HT2C receptor agonists can rescue PPI deficits induced by psychomimetics (Marquis et al., 2007). Thus, the multi-pharmacological profile of SCH 23390 in amelioration of IVM-induced PPI deficits cannot be disregarded. Moreover, although the ventral striatum [which comprises of nucleus accumbens (NAc) core] is ascertained as an important site for PPI mediation; this behavior is regulated by the cortico-pallido-striatal-thalamic circuitry (Wan et al., 1995; Wan and Swerdlow, 1996; Swerdlow et al., 2008, 2011). Considering that DA receptor agonists induce PPI dysfunction and DA receptor antagonists reverse PPI deficits, it is presumably thought that increased DAergic function in the NAc (Swerdlow et al., 2001a), leads to inhibition of GABAergic neurons projecting toward the ventral pallidum (VP), which has a tonic control over the pedunculopontine nucleus (PPTg), which mediates PPI function (Fendt et al., 2001). Thus, disruption of underlying signaling cascades in any of these brain structures of this complex circuitry could possibly be linked to the antagonistic interaction between IVM and SCH 23390 which warrants further investigation. Detailed delineation of signaling pathways within the PPI circuitry maybe necessitated in elucidating the discrepancy between SCH 23390 and SKF 82958 in regulation of IVM-mediated behavioral effects.

In addition to DARPP-32, we also evaluated the effects of IVM on other signaling molecules that may contribute to alterations in sensorimotor gating such as nNOS and its upstream regulator, CaMKIIα. CaMKIIα can phosphorylate nNOS at Ser897, leading to reduction in functional activity (Hayashi et al., 1999; Komeima et al., 2000). IVM decreased CaMKIIα phosphorylation in a D2-dependent manner since its effects were reversed by raclopride, but not by SCH 23390. Nevertheless, SCH 23390 was able to prevent IVM from further decreasing CaMKIIα phosphorylation suggesting that SCH 23390 might be reversing IVM-mediated effects on signaling pathways upstream of CaMKIIα phosphorylation. Interestingly, at a lower dose (5 mg/kg), IVM significantly potentiated the effects of SKF 82958 on CaMKIIα phosphorylation. A mechanistic explanation for this outcome is the synergistic interaction between IVM and SKF 82958 in DARPP-32 phosphorylation which could lead to up-regulation of CaMKIIα phosphorylation via inhibition of protein phosphotase-1 (PP-1) (Strack et al., 1997; Shioda and Fukunaga, 2017). Overall, these findings suggest that the dopaminergic control of IVM over CaMKIIα phosphorylation can be dose-dependent. While there is no direct evidence that links CaMKIIα to PPI regulation; results from our investigation mirror PPI disruptive agents such as indirect DA agonists (methamphetamine) (Suemaru et al., 2000) and *N*-Methyl-D-aspartate receptor (NMDAR) antagonists (PCP, ketamine) (Molteni et al., 2008; Cui et al., 2009) that have been reported to decrease CaMKIIα phosphorylation in mice and that this effect was fully restored upon D1 or D2 receptor antagonism. Additionally, CaMKIIα has been implicated in information processing (Coultrap and Bayer, 2012) and pathophysiology of psychiatric disorders characterized by cognitive abnormalities (Frankland et al., 2008; Yamasaki et al., 2008; Matsuo et al., 2009). If PPI deficiency can precede fragmentation of cognitive function (McGhie and Chapman, 1961); then alterations in CaMKIIα activity can be linked to PPI deficits in the clinical population.

On account of the significant reduction in CaMKIIα phosphorylation, IVM tended to reduce the phosphorylation of its downstream target, nNOS. Although raclopride, but not SCH 23390, was able to reverse IVM-mediated decrease in CaMKIIα phosphorylation; the pharmacological blockade of both D1 and D2 receptors significantly reversed IVM-mediated changes in nNOS phosphorylation. These results suggest that CaMKIIα could potentially be involved upstream in the effects of D2, but not D1, receptors on IVM-induced changes in nNOS phosphorylation. Although D1 receptor activation can enhance nNOS activity via NMDARs-mediated Ca^2+^ currents which involves CaMKIIα (Cepeda et al., 1998; Lee et al., 2010), stimulation of D1 receptors can also lead to enhanced phosphorylation of nNOS at Ser897 via PKA activation (Missale et al., 1998; Neve et al., 2004) or protein kinase C (PKC) activation (Felder et al., 1989; Undie and Friedman, 1990) which could ultimately result in reduced nNOS activity (Bredt et al., 1992; Dinerman et al., 1994; Lee et al., 2010). In further support of the argument that CaMKIIα may not be involved in the interaction between P2X4Rs and D1 receptors in nNOS phosphorylation, IVM potentiated the effects of SKF 82958 in regulation of CaMKIIα phosphorylation but decreased SKF 82958-induced increase in nNOS phosphorylation.

The ability of IVM to increase nNOS activity (via reduced phosphorylation of nNOS at Ser897) can lead to increased DARPP-32 phosphorylation at Thr34 via the NO/cGMP/guanyl cyclase (GC)/protein kinase G (PKG) signaling cascade (Tsou et al., 1993; Nishi et al., 2005; Threlfell and West, 2013). Additionally, the ability of IVM to dampen SKF 82958-induced decrease in nNOS phosphorylation may provide a mechanistic explanation for the synergy between IVM and SKF 82958 in regulation of DARPP-32 phosphorylation. This could represent a potential mechanism underlying IVM-mediated increased DARPP-32 phosphorylation considering that P2X4Rs are LGICs localized on GABAergic interneurons (Amadio et al., 2007) that are enriched in nNOS (Bredt et al., 1990, 1991) and have been reported to play a role in modulation of NOS activity via the cGMP/GC/PKG pathway (Yang et al., 2015). Hence, it is more logical to assume that cGMP/PKG pathway is involved upstream of P2X4R-mediated effects on DARPP-32 phosphorylation rather than the canonical cAMP/PKA activity which is regulated by G-coupled protein receptors (GPCRs) (Pierce et al., 2002). Based on previous evidence that have supported an interaction between DA neurotransmission and nNOS activity (Sammut et al., 2006, 2007; Hoque et al., 2010; Hoque and West, 2012), the reversal of IVM-mediated decrease in nNOS phosphorylation by DA receptor antagonists may underlie their antagonistic interaction with IVM in regulation of DARPP-32 phosphorylation. However, future investigations testing the effects of IVM on NO and cGMP levels in presence of dopaminergic drugs would be warranted to confirm this hypothesis.

In contrast to CaMKIIα, there is a growing body of evidence that supports role of nNOS in PPI regulation in mice (Klamer et al., 2004; Salum et al., 2006; Fejgin et al., 2008; Issy et al., 2009, 2014). The ability of IVM to decrease nNOS phosphorylation at Ser897 and induce PPI deficits is in corroboration with current theory that increased NO signaling leads to PPI deficits. NOS inhibitors have been observed to attenuate PPI deficits mediated by DA receptor activation (amphetamine, methylphenidate) (Issy et al., 2009) or NMDARs antagonism (PCP) (Klamer et al., 2004; Fejgin et al., 2008) From a disease standpoint, multiple preclinical and clinical findings have linked increased NO signaling to pathophysiology of psychiatric disorders characterized by sensorimotor gating deficits including schizophrenia (Bernstein et al., 2001; Yao et al., 2004; Bernstein et al., 2005; Lauer et al., 2005; Nasyrova et al., 2015). Genetic deficiency of nNOS generates a mouse model that exhibits behavioral and neurochemical abnormalities that are reminiscent of psychiatric disorders (Tanda et al., 2009). Increased nNOS activity has been detected in several brain regions in schizophrenia (Karson et al., 1996; Bernstein et al., 2001; Baba et al., 2004). Furthermore, genetic polymorphisms in nNOS have been detected as risk factor for schizophrenia (Reif et al., 2006; Rovny et al., 2018). Overall, based on findings from previous reports and current investigation, the regulation of nNOS activity can contribute to dopaminergic control of IVM-mediated PPI deficits and P2X4Rs-mediated increase in nNOS activity can represent a novel mechanism underlying psychiatric disorders characterized by sensorimotor gating perturbations especially schizophrenia.

Deficits in PPI have been linked to a wide spectrum of “perceptual” disorders including schizophrenia (Braff et al., 1978, 1999; Braff, 1993; Kumari et al., 1999, 2000), bipolar disorder (Perry et al., 2001), obsessive–compulsive disorder (Swerdlow et al., 1993) and Tourette’s syndrome (Swerdlow et al., 2001b) as well as to autism spectrum disorders such as autism (Perry et al., 2007) and Fragile X syndrome (Frankland et al., 2004). Mutant mouse models for susceptible genes that have been suggested to be involved in the pathophysiology of aforementioned diseases exhibit PPI dysfunction (Ralph et al., 2001; Duncan et al., 2004; Kinkead et al., 2005; Tanaka et al., 2006; Ohgake et al., 2009; Lipina et al., 2010; Willi et al., 2010; Vuillermot et al., 2011), making PPI a reliable endophenotype in the genetic studies of neuropsychiatric disorders (Braff et al., 2007; Powell et al., 2012). Moreover, several of the clinical typical and atypical anti-psychotics such as haloperidol, risperidone, clozapine, olanzapine, quetiapine have been demonstrated to ameliorate PPI deficits in genetic and pharmacological models of such diseases (Swerdlow and Geyer, 1993; Bast et al., 2001; Geyer et al., 2001; Kumari and Sharma, 2002; Levin et al., 2007). Thus, PPI has been elucidated as a useful behavioral assay in investigating molecular mechanisms of psychiatric diseases as well as screening of potential antipsychotic drugs. The ability of IVM to disrupt PPI function via P2X4R potentiation (Bortolato et al., 2013) as well as the reduced PPI function in the P2X4R KO mouse model (Wyatt et al., 2013) suggests a role for P2X4Rs in pathophysiology of psychiatric disorders characterized by sensorimotor gating perturbations. Additionally, our findings also suggest a role for DA neurotransmission in altering IVM-mediated effects on sensorimotor gating. Considering the link between dysregulated dopaminergic function and sensorimotor gating abnormalities in psychiatric diseases (Swerdlow et al., 1986; Braff and Geyer, 1990; Swerdlow and Geyer, 1993; Braff et al., 2001) has been well consolidated over past decades, our preclinical studies suggest a role for P2X4Rs in pathophysiology of psychiatric diseases. Moreover, we have also demonstrated that IVM can induce dysregulation in signaling molecules including DARRP-32, CaMKIIα and nNOS, all of which have been previously linked to psychiatric diseases characterized by sensorimotor gating abnormalities (Albert et al., 2002; Bernstein et al., 2005; Ishikawa et al., 2007; Frankland et al., 2008; Nasyrova et al., 2015; Wang et al., 2017). However, further investigations at the clinical level would be warranted to link *p2rx4* gene mutations (leading to aberrations in functioning) with neuropsychiatric disorders in order to ascertain such a role for P2X4Rs in patients diagnosed with psychiatric illnesses. Until date, single nucleotide polymorphisms in human *p2rx4* gene have been only linked to hypertension and age-related macular degeneration (Caseley et al., 2014).

Although the findings from our current investigation provide novel mechanistic insights into role of P2X4Rs in regulation of sensorimotor gating, there are certain limitations of our study that needs to be acknowledged. First, we only identified the effects of IVM in regulation of signaling molecules in one brain region that is part of a complex neural circuitry that regulates PPI function (Swerdlow et al., 2001a, 2008). Hence, future investigations that will test the impact of dopaminergic drugs on IVM-mediated changes in DARPP-32, CaMKIIα and nNOS phosphorylation in other brain regions integral to PPI circuitry including medial prefrontal cortex (mPFC), ventral hippocampus and basolateral amygdala would be warranted. Elucidation of the interaction between P2X4Rs and DA receptors in these specified brain regions would provide a holistic overview of role of P2X4Rs in mechanisms of sensorimotor gating. Another major limitation of our study is the absence of translational applicability due to lack of specific P2X4R antagonists. Based on our results with IVM, antagonism of P2X4R function would alleviate sensorimotor gating deficits and represent a novel therapeutic strategy. An alternative approach is to use viral vectors that would specifically knockdown P2X4R expression in a brain region critical for PPI regulation such as the NAc, mPFC or ventral hippocampus. Lastly, we did not establish a link between DARPP-32, CaMKIIα or nNOS and the dopaminergic control of IVM-mediated PPI deficits. Hence, future experiments would include testing the impact of interaction between P2X4Rs and DA receptors on PPI regulation upon genetic ablation of these signaling molecules by viral vectors or in genetic knockout mouse models. Such investigations would be pivotal for establishing a role for DARPP-32, CaMKIIα and nNOS in IVM-mediated PPI deficits.

Overall, the current investigation provides novel insights into a potential interaction between P2X4Rs and DA receptors in modulation of PPI and that perturbation of this interaction could lead to manifestation of sensorimotor gating deficits and subsequent cognitive fragmentation. Most importantly, our findings support the development of P2X4R antagonists as potential anti-psychotics for treatment of diseases linked to DA-dependent sensorimotor gating dysfunction.

## Data Availability

The datasets generated for this study are available on request to the corresponding author.

## Ethics Statement

All experiments were undertaken as per guidelines established by the National Institutes of Health (NIH) and approved by the Institutional Animal Care and Use Committee (IACUC) at the University of Southern California, Los Angeles, CA, United States.

## Author Contributions

SK designed the research study, performed the experiments, analyzed the data, and wrote the manuscript. LA, MJ, and DD reviewed and edited the manuscript.

## Conflict of Interest Statement

DD and LA are inventors on a patent for the use of ivermectin for treatment of alcohol use disorders. The remaining authors declare that the research was conducted in the absence of any commercial or financial relationships that could be construed as a potential conflict of interest.
