# Supplementary material for: Dopamine Receptor Blockade Attenuates Purinergic P2X4 Receptor-Mediated Prepulse Inhibition Deficits and Underlying Molecular Mechanisms
Source: Front Cell Neurosci. 2019 Jul 23;13:331. doi: 10.3389/fncel.2019.00331 (PMC6664007; doi:10.3389/fncel.2019.00331)
Supplement: Supplementary file 1 [file Data_Sheet_1.pdf]

DOPAMINE RECEPTOR BLOCKADE ATTENUATES PURINERGIC P2X4 RECEPTOR-MEDIATED PREPULSE INHIBITION DEFICITS AND UNDERLYING MOLECULAR MECHANISMS

Sheraz Khoja[1]; Liana Asatryan[1]; Michael W. Jakowec[2]; Daryl L. Davies[1*]

[1]Titus Family of Clinical Pharmacy, School of Pharmacy, University of Southern California, Los Angeles, CA 90089

[2]Department of Neurology, Keck School of Medicine, University of Southern California, Los Angeles, CA 90033

*Correspondence:

Dr. Daryl L. Davies
Email ID: ddavies@usc.edu

Keywords: ivermectin, P2X4 receptors, dopamine receptors, prepulse inhibition, schizophrenia, DARPP-32

Number of words in abstract: 350
Number of words total: 7190
Number of figures: 6
Number of tables: 0
Number of figures in supplemental section: 7

Fig S1

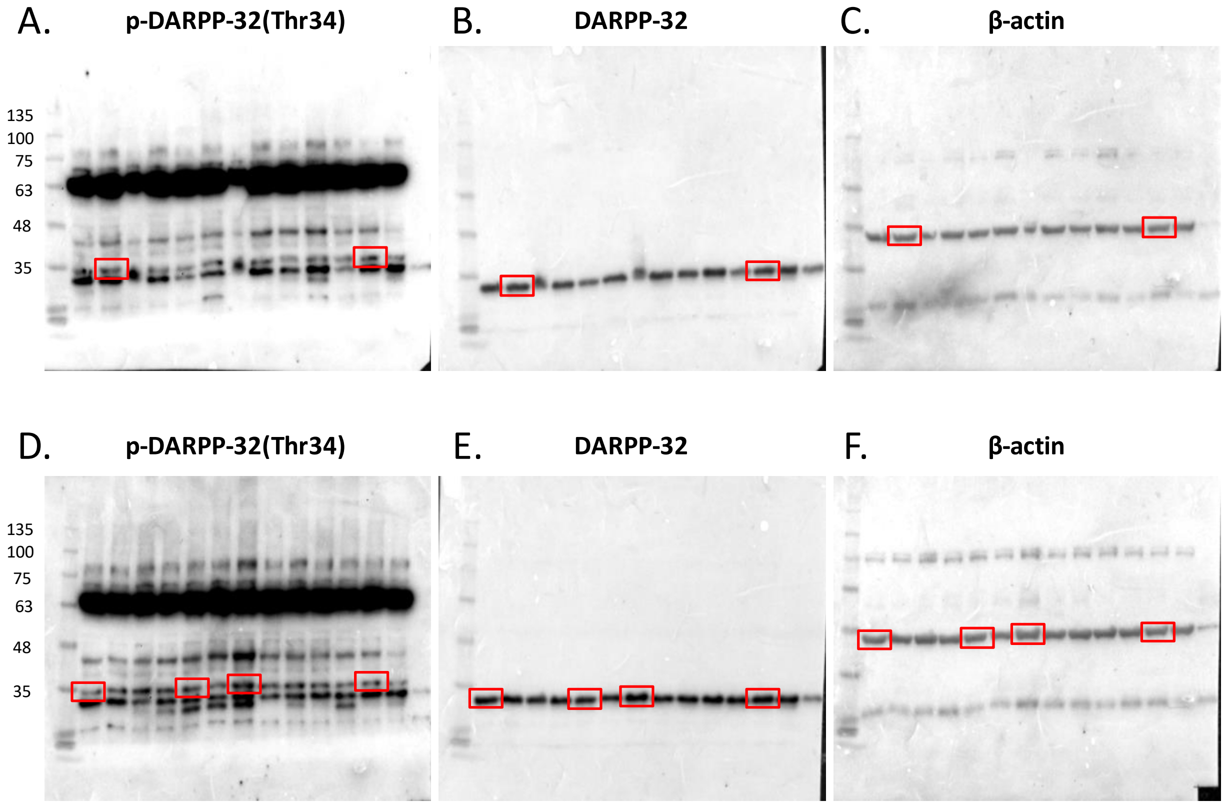

Figure S1: Full scan of original blots for gel # 1 [p-DARPP-32(Thr34) (A), total DARPP-32(B), β-actin (C)] and gel # 2 [p-DARPP-32(Thr34) (D), total DARPP-32 (E), β-actin (F)] for the immunoblotting experiment with IVM (10 mg/kg) and DA receptor antagonists for Figure 3. Blots that were cropped and spliced together from different gels or non-consecutive lanes are shown in red boxes.

Fig S2

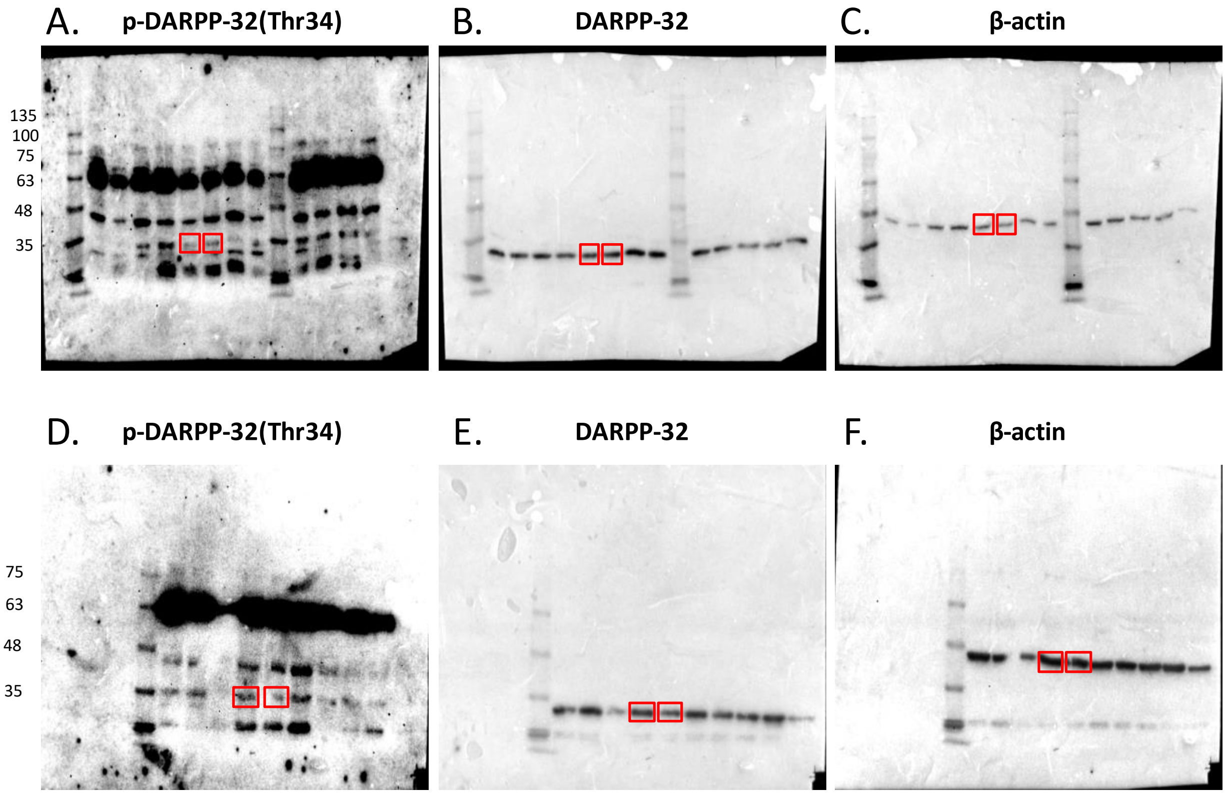

Figure S2: Full scan of original blots for gel # 1 [p-DARPP-32(Thr34) (A), total DARPP-32 (B), β-actin (C)] and gel # 2 [p-DARPP-32(Thr34) (D), total DARPP-32 (E), β-actin (F)] for the immunoblotting experiment with IVM (5 mg/kg) and SKF 82958 for Figure 3. Blots that were cropped and spliced together from different gels or from non-consecutive lanes are shown in red boxes.

Fig S3

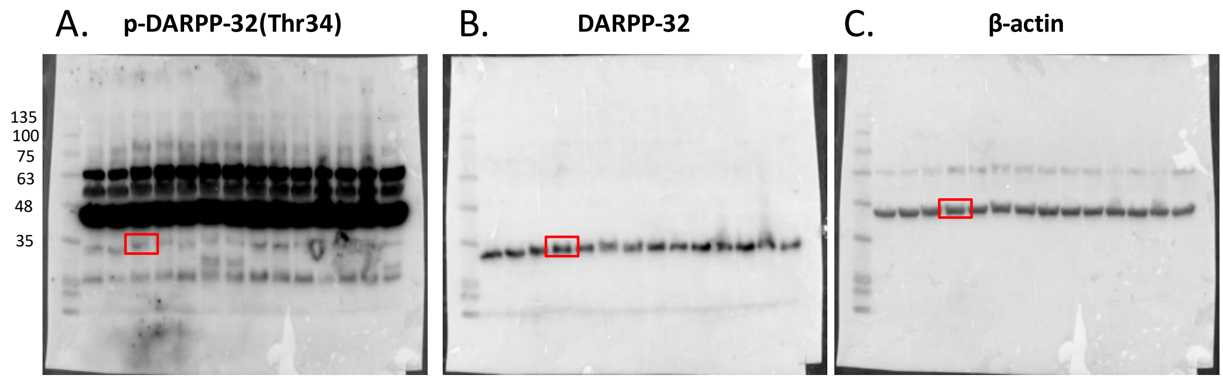

Figure S3: Full scan of original blots for gel # 1 [p-DARPP-32 (Thr34) (A), total DARPP-32 (B), β-actin (C)] for the immunoblotting experiment with IVM (5 mg/kg) and SKF 82958 for Figure 3. Blots that were cropped and spliced together from different gels or non-consecutive lanes are shown in red boxes.

Fig S4

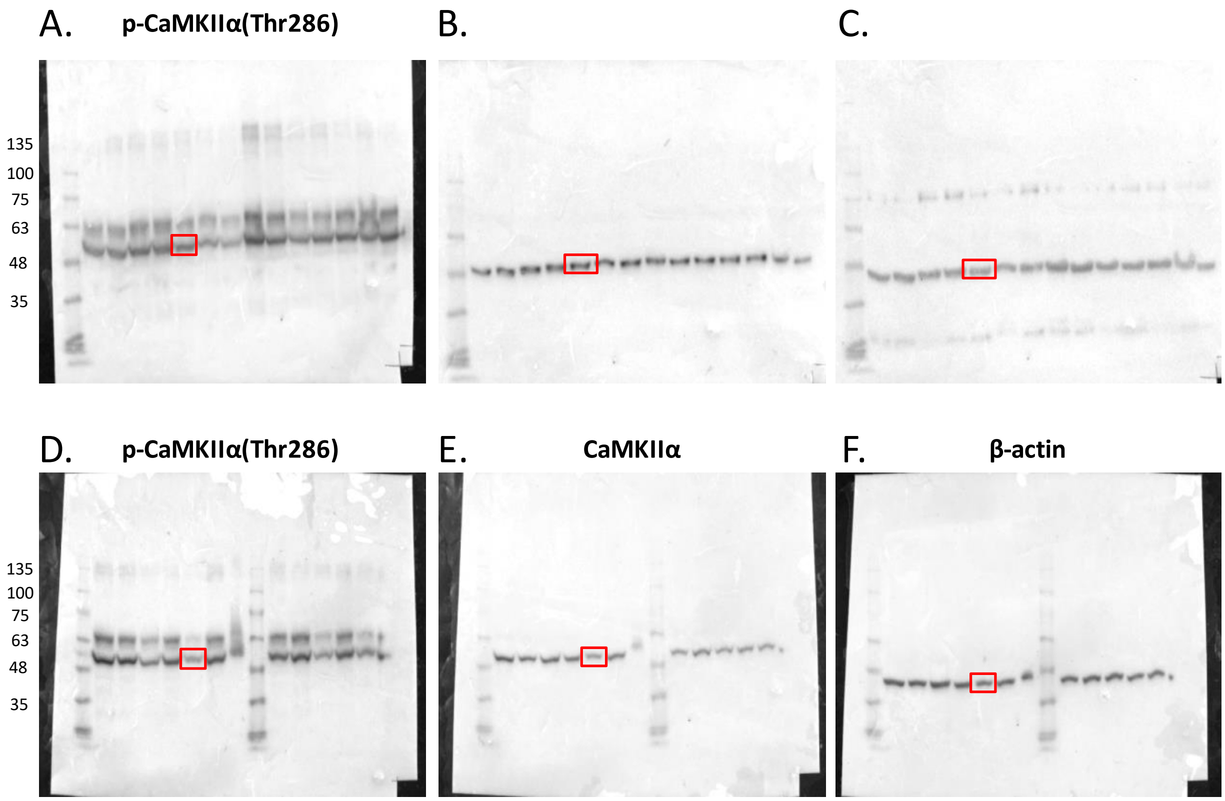

Figure S4: Full scan of original blots for gel # 1 [p-CaMKIIα (Thr286) (A), total CaMKIIα (B), β-actin (C)] and gel # 2 [p-CaMKIIα (Thr286) (D), total CaMKIIα (E), β-actin (F)] for the immunoblotting experiment with IVM (10 mg/kg) and DA receptor antagonists for Figure 4. Blots that were cropped and spliced together from different gels or non-consecutive lanes are shown in red boxes.

Fig S5

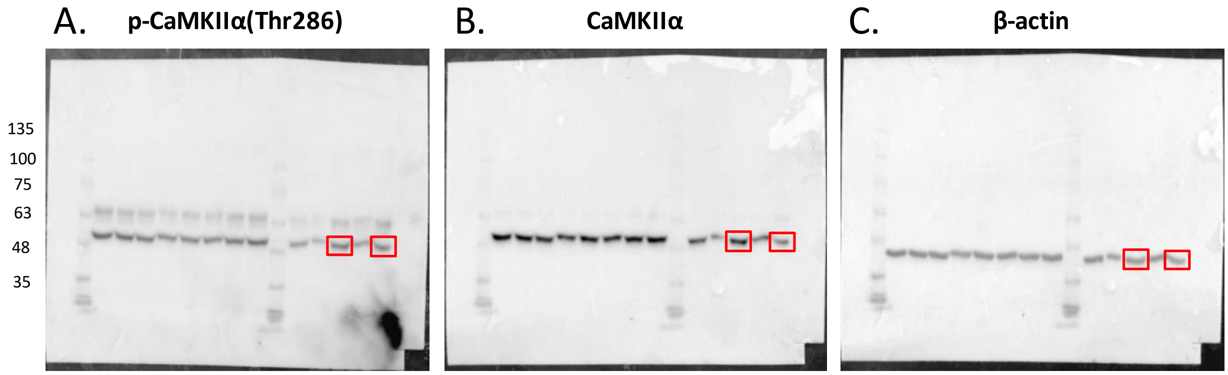

Figure S5: Full scan of original blots for gel # 1 [p-CaMKIIα (Thr286) (A), total CaMKIIα (B), β-actin (C)] for the immunoblotting experiment with IVM (5 mg/kg) and SKF 82958 for Figure 4. Blots that were cropped and spliced together from different gels or non-consecutive lanes are shown in red boxes.

Fig S6

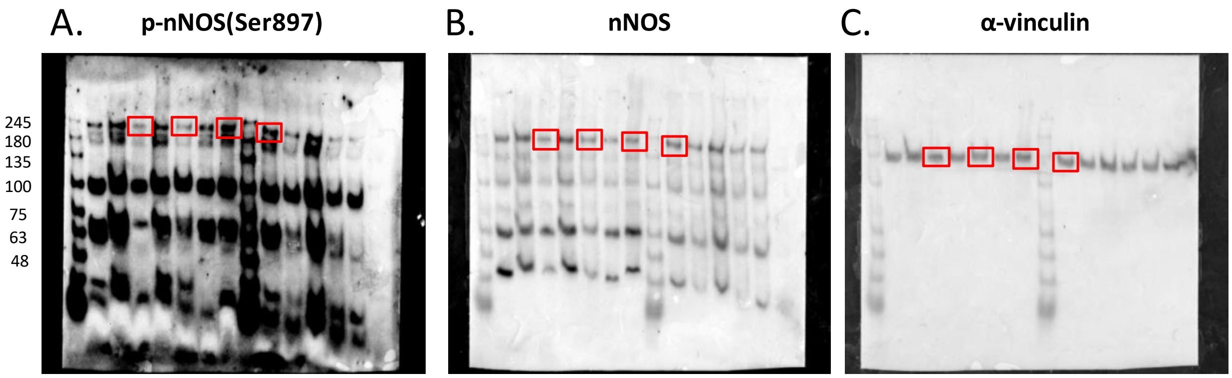

Figure S6: Full scan of original blots for gel # 1 [p-nNOS (Ser897) (A), total nNOS (B), α-vinculin (C)] for the immunoblotting experiment with IVM (10 mg/kg) and DA receptor antagonists for Figure 5. Blots that were cropped and spliced together from different gels or non-consecutive lanes are shown in red boxes.

Fig S7

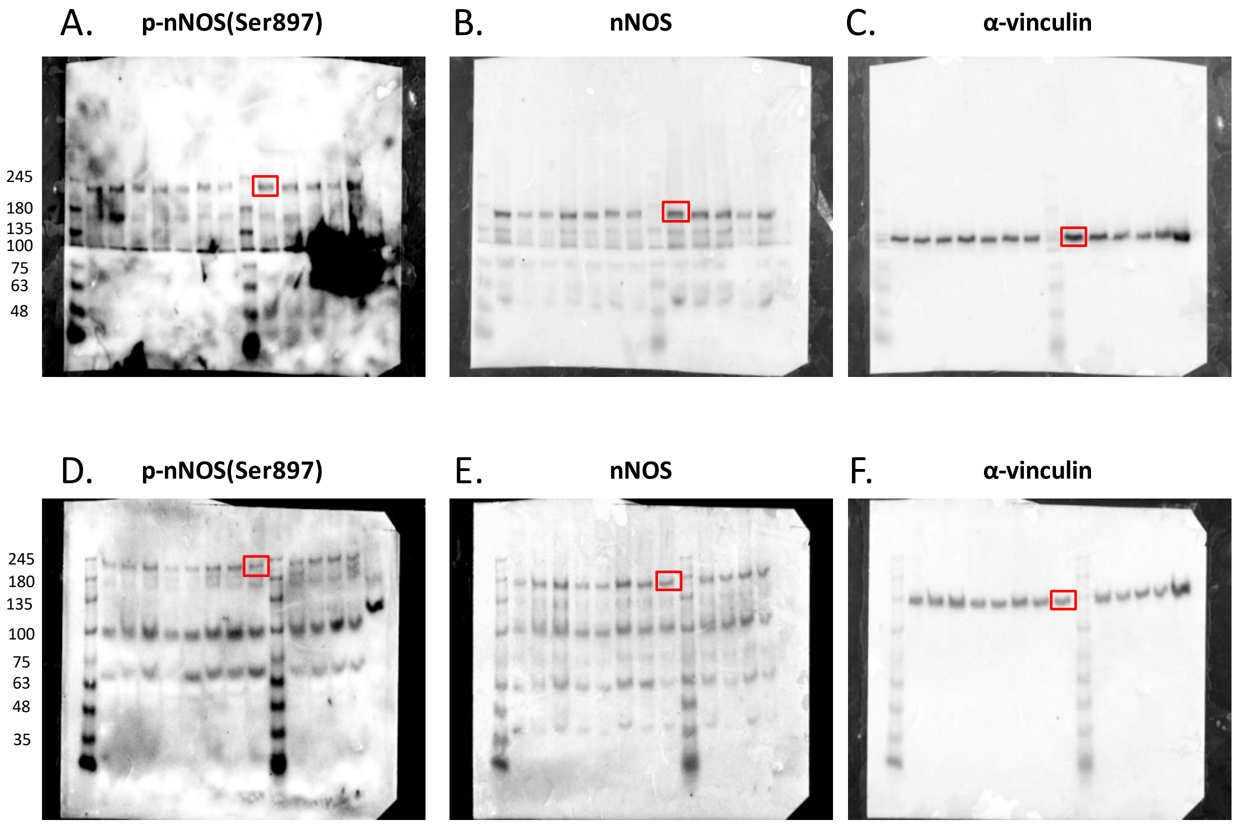

Figure S7: Full scan of original blots for gel # 1 [p-nNOS (Ser897) (A), total nNOS (B), α-vinculin (C)] and gel # 2 [p-nNOS (Ser897) (D), total nNOS (E), α-vinculin (F)] for the immunoblotting experiment with IVM (10 mg/kg) and DA receptor antagonists for Figure 5. Blots that were cropped and spliced together from different gels or non-consecutive lanes are shown in red boxes.
